# Variation in Yield Responses to Elevated CO_2_ and a Brief High Temperature Treatment in Quinoa

**DOI:** 10.3390/plants6030026

**Published:** 2017-07-05

**Authors:** James A. Bunce

**Affiliations:** Crop Systems and Global Change Laboratory, United States Department of Agriculture, 10300 Baltimore Avenue, Beltsville, MD 20705-2350, USA; buncejames49@gmail.com; Tel.: +1-301-504-7629

**Keywords:** quinoa, elevated CO_2_, high temperature stress, photosynthesis, harvest index, seed yield

## Abstract

Intraspecific variation in crop responses to global climate change conditions would provide opportunities to adapt crops to future climates. These experiments explored intraspecific variation in response to elevated CO_2_ and to high temperature during anthesis in *Chenopodium quinoa* Wild. Three cultivars of quinoa were grown to maturity at 400 (“ambient”) and 600 (“elevated”) μmol·mol^−1^ CO_2_ concentrations at 20/14 °C day/night (“control”) temperatures, with or without exposure to day/night temperatures of 35/29 °C (“high” temperatures) for seven days during anthesis. At control temperatures, the elevated CO_2_ concentration increased the total aboveground dry mass at maturity similarly in all cultivars, but by only about 10%. A large down-regulation of photosynthesis at elevated CO_2_ occurred during grain filling. In contrast to shoot mass, the increase in seed dry mass at elevated CO_2_ ranged from 12% to 44% among cultivars at the control temperature. At ambient CO_2_, the week-long high temperature treatment greatly decreased (0.30 × control) or increased (1.70 × control) seed yield, depending on the cultivar. At elevated CO_2_, the high temperature treatment increased seed yield moderately in all cultivars. These quinoa cultivars had a wide range of responses to both elevated CO_2_ and to high temperatures during anthesis, and much more variation in harvest index responses to elevated CO_2_ than other crops that have been examined.

## 1. Introduction

The yearly mean concentration of carbon dioxide in the atmosphere has increased from about 320 μmol·mol^−1^ in 1965 to about 400 μmol·mol^−1^ currently, and continues to increase rapidly. Research has shown that concentrations above the current ambient concentration generally increase the growth and yield of C_3_ crop species, and could stimulate future crop yields, unless other changes in climate, such as rising temperatures or altered precipitation, interfere. Intraspecific variation in the stimulation in yield at elevated carbon dioxide concentrations has been detected in many crops species, including barley [1], common bean [2], cowpea [3], soybean [4,5], wheat [6,7], oats [8], rapeseed [1,9], and rice [10]. Identification of traits associated with larger yield increases at elevated carbon dioxide would be desirable for breeding cultivars better able to exploit the rising carbon dioxide concentration. In most cases, reasons for cultivar differences in yield response to elevated carbon dioxide, beyond differential increases in seed number, have not been identified [11]. Harvest index, defined as the ratio of the economic product relative to the total aboveground biomass at maturity, generally decreases slightly or is unchanged at elevated CO_2_ in crops where the economic product results from sexual reproduction [12,13,14].

Rising mean temperature and increased frequency of extreme high temperature events may reduce crop yields [15,16,17,18,19]. Many annual seed-producing crops are grown in regions where temperatures during reproductive development are near or even above the optimum for seed yield [19], and increases in temperature tend to decrease yields because of high temperature stress, in spite of increasing the growing season length. Despite predictions that elevated CO_2_ might mitigate reductions in yield caused by high temperatures based on increasing the optimum temperature for photosynthesis [19,20], several studies in various crop species have found that elevated CO_2_ exacerbated yield reductions caused by high temperatures [21,22,23,24,25,26,27,28,29]. With the exception that elevated CO_2_ may increase tissue temperatures by reducing stomatal conductance and transpiration [26], reasons why elevated CO_2_ may exacerbate yield reductions caused by high temperatures remain largely unknown. There is little information about differences among cultivars in response to high temperature and elevated CO_2_ treatments [28]. 

Quinoa (*Chenopodium quinoa* Willd) is an annual grain crop native to the Andes of South America, with Peru and Bolivia currently being the largest producers. It is increasing in popularity because of its high nutritional quality. It has been studied for variation in adaptation to salinity, drought, and elevation [30,31,32,33,34], but not with regard to climate change factors. In this study, three cultivars were studied for responses to elevated CO_2_ and to high temperature stress during anthesis, which is typically the phase of development in which seed yield is most sensitive to high temperature stress [18]. 

## 2. Results

The beginning of anthesis of main stem flowers occurred at 40 to 44 days after planting of seeds, depending upon the cultivar, and CO_2_ concentration had no effect on this timing (not shown). Most main stem leaves had senesced and seeds were mature at about 90 days after planting, except that the high temperature treatment at the lower CO_2_ concentration resulted in a slower progression of anthesis up the main stem in Salcedo, which prolonged seed filling and delayed maturity by about 10 days. The failure of seed development resulting from the high temperature treatment was evident in Cherry Vanilla grown at the lower CO_2_ concentration, but not in other cases. In Cherry Vanilla with the high temperature treatment, there was no flower abortion, but flowers often produced no seeds.

Leaf photosynthetic rates were very similar for the three cultivars under all conditions (Table 1). Increasing CO_2_ from 400 to 600 μmol·mol^−1^ increased leaf photosynthesis of plants grown at the lower CO_2_ concentration by about 14% at 20 °C, at the growth photosynthetic photon flux density (PPFD) (Table 1). At 35 °C, the increase was about 25%. Prior to anthesis, and during the high temperature treatment, there was little difference in photosynthesis between plants grown at the two CO_2_ concentrations when measured at the higher CO_2_ (Table 1), indicating no significant down-regulation of photosynthesis due to growth at elevated CO_2_ at these stages of development. However, after the stress period, and during grain filling, there was no difference in photosynthesis between plants grown at 400 and 600 μmol·mol^−1^ CO_2_ measured at their growth CO_2_ and PPFD conditions, while plants grown at the lower CO_2_ concentration had higher rates when measured at 600 μmol·mol^−1^ (Table 1). This is indicative of the down-regulation of photosynthesis during the grain filling period, resulting from growth at the elevated CO_2_ concentration. There was no after-effect of the heat stress on leaf photosynthetic rates in any cultivar (Table 1).

**Figure 1:**
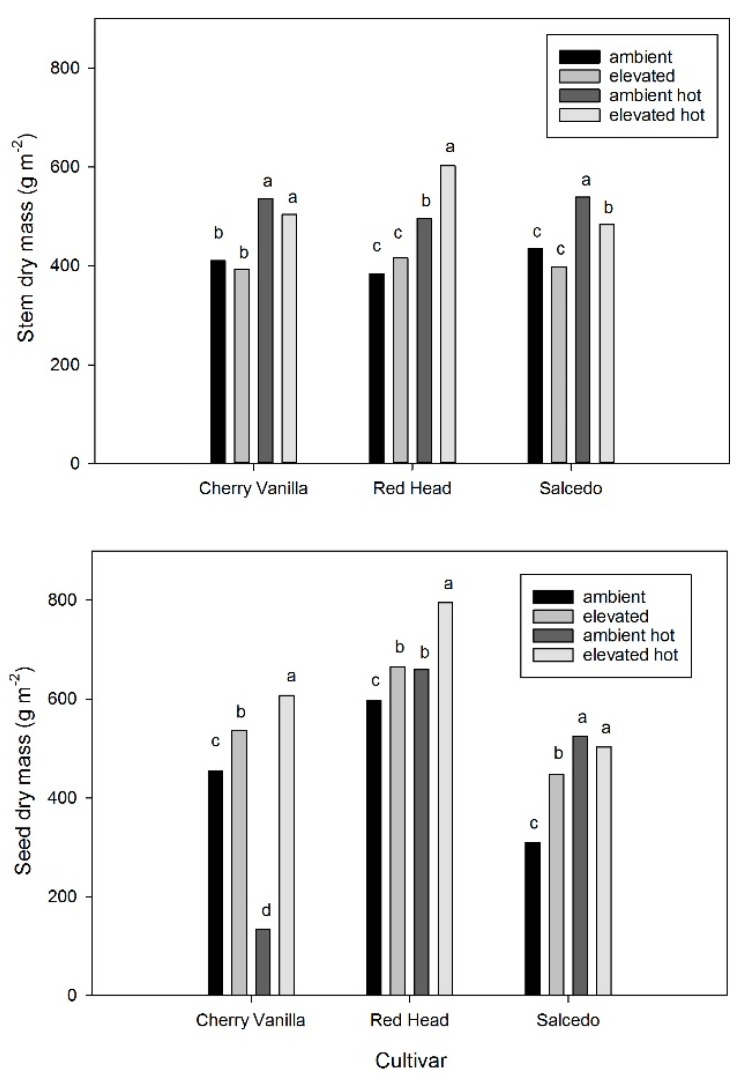
Stem and seed dry mass responses of three cultivars of quinoa to ambient (400 μmol·mol^−1^) or elevated (600 μmol·mol^−1^) CO_2_ concentrations, with or without a high temperature treatment for 7 days beginning at anthesis. The control growth day/night temperatures were 20/14 °C, and the high temperature treatment was 35/29 °C. Within cultivars, different letters indicate significant differences in mean values, based on analysis of variance (ANOVA).

**Figure 2:**
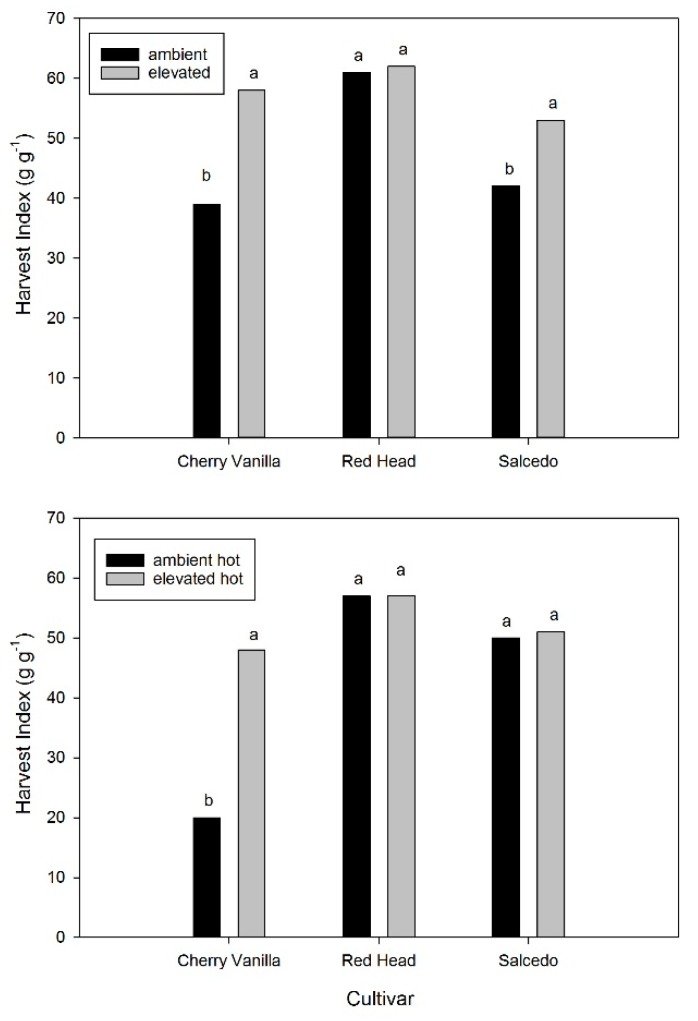
Harvest index (seed dry mass/total aboveground dry mass) at final harvest for three cultivars of quinoa grown at ambient (400 μmol·mol^−1^) or elevated (600 μmol·mol^−1^) CO_2_ concentrations, with or without a high temperature treatment for 7 days beginning at anthesis. The control growth day/night temperatures were 20/14 °C, and the high temperature treatment was 35/29 °C. Within cultivars and temperature treatments, different letters indicate significant differences in mean values, based on ANOVA.

Seed yield was affected by the CO_2_ and high temperature treatments in a complex manner, as indicated by a significant CO_2_ × T × cultivar interaction (*p* = 0.023). At the control temperature, stem dry mass at maturity was not increased significantly by elevated CO_2_ in any cultivar (Figure 1). In contrast to the lack of increase in stem dry mass, seed dry mass was increased by elevated CO_2_ in all three cultivars, ranging from 12% in Red Head to 44% in Salcedo (Figure 1). Total shoot dry mass was increased by the high CO_2_ treatment in all cultivars, but only by 8% to 13%. Harvest index was significantly increased by the elevated CO_2_ treatment in both Salcedo and Cherry Vanilla for the control temperature treatment, but unchanged in Red Head (Figure 2). 

The high temperature at anthesis treatment produced a wider range of responses to elevated CO_2_ than had occurred at the control temperature. With the high temperature treatment, elevated CO_2_ greatly increased seed mass in Cherry Vanilla, moderately increased seed mass in Red Head, and did not change seed mass in Salcedo (Figure 1). Stem mass was increased by the high temperature treatment in all cultivars and for both CO_2_ levels (Figure 1). For the high temperature stress regime, stem mass was increased by elevated CO_2_ only in the case of Red Head (Figure 1). At ambient CO_2_, the high temperature treatment either decreased (0.30 × control, Cherry Vanilla) or increased (1.70 × control, Salcedo) seed yield, or had little effect (Red Head), depending on the cultivar (Figure 1). In contrast, at elevated CO_2_, the high temperature treatment increased seed yield by 12% to 19% in all cultivars (Figure 1). With the high temperature treatment, the harvest index was greatly increased by elevated CO_2_ in Cherry Vanilla, but unchanged in Red Head and Salcedo (Figure 2). The mean mass per seed was unaffected by either the elevated CO_2_ treatment or the heat stress treatment in any of the cultivars. 

## 3. Discussion

The decrease in photosynthesis during the high temperature treatment was less at elevated than at ambient CO_2_ concentrations (Table 1), consistent with expectations based on Rubisco thermal kinetics [35]. The similar, small increase in total aboveground biomass at harvest of all cultivars at the control temperature was consistent with the large down-regulation of photosynthesis at elevated CO_2_, which occurred during grain filling in all the cultivars. However, relationships between photosynthetic and growth responses for the high temperature treatments were unclear, based on the very similar photosynthetic responses to the treatments among the three cultivars and their divergent total biomass responses to the treatments.

The significant differences in yield response among these three cultivars of quinoa to the elevated CO_2_ and high temperature treatments offer promise for adapting this crop to changes in climate. At the control temperature, two of the cultivars, Cherry Vanilla and Salcedo, had substantially increased yield at elevated CO_2_ because of increased harvest index. For other crops where agronomic yield results from sexual reproduction, increases in harvest index with elevated CO_2_ have not generally been found, with slight decreases in harvest index being very common [12,13,14,24]. This is despite the generalization that elevated CO_2_ stimulates flower production and/or reduces flower and seed abortion by increasing whole plant photosynthate supply [36]. Gomez et al. [37] found that the harvest index in quinoa can be quite flexible in response to the manipulation of gibberellin synthesis. For the two quinoa cultivars which had an increased harvest index at elevated CO_2_ in this experiment, it was not investigated whether the increased harvest index resulted from increased numbers of flowers or reduced losses of reproductive structures. However, consistent with other grain crops [36], seed size was unaffected by the elevated CO_2_ treatment in all of the cultivars.

The lack of decrease in total seed mass at elevated CO_2_ when plants were exposed to the high temperature treatments observed here in quinoa (significant increases in two cultivars, Figure 1) contrasts with the response found in several studies with other species. For example, in rice and wheat, even small increases in temperature in combination with elevated CO_2_ decreased yields in free air carbon dioxide enrichment (FACE) experiments [22]. The same decrease in yield due to high temperatures for plants at elevated CO_2_ also occurred in soybean [26] and maize [27] in FACE experiments. These results are consistent with several earlier studies using other exposure systems [24]. Wang et al. [28] showed that brief high temperatures at anthesis caused more yield reductions than did longer-term, milder high temperature treatments in rice, and that yield reductions caused by high temperatures in both cases were greater at elevated than at ambient CO_2_. Prassad et al. [25] showed in glasshouse experiments with sorghum that elevated CO_2_ resulted in higher tissue temperatures during heat stress than occurred at ambient CO_2_, which could potentially explain why elevated CO_2_ made crops more sensitive to heat stress. However, in contrast to these responses, anecdotal observations on wheat indicated that elevated CO_2_ could reduce the negative impact of heat waves on yield [38]. Additionally, Ferris et al. [39] found that brief high temperature treatments which decreased soybean seed yield at ambient CO_2_ increased yield at elevated CO_2_ in glasshouse experiments, evidenced by a significant CO_2_ × T interaction. These observations as well as the results presented here for quinoa provide hope that cultivars may be found in several crops in which rising atmospheric CO_2_ concentrations may mitigate the negative effects of high temperature stress on yield.

## 4. Materials and Methods

Three open pollinated cultivars of quinoa, *Chenopodium quinoa* Willd. Red Head, Cherry Vanilla, and Salcedo, were grown from seed in indoor controlled environment chambers. The Red Head and Cherry Vanilla cultivars were developed in the USA. from Peruvian germplasm, and Salcedo is a commercial cultivar from Peru. The cultivars from the USA. have reduced yields in hot climates, by informal reputation. Plants were grown with 12 h of light per day at 1000 μmol·m^−2^·s^−1^ PPFD from a mixture of high pressure sodium and metal halide lamps in Environmental Growth Chambers (M-18 Chambers). Temperature control was ±0.3 °C. Initial day/night temperatures were 20/14 °C, with a dew point temperature of 10 °C. Plants were grown with one plant per pot in 25 cm diameter plastic pots filled with vermiculite, and flushed to the drip point once or twice per day with a complete nutrient solution containing 14.5 mM nitrogen. The chemical composition of the nutrient solution is given in Shimono and Bunce [40], except that for quinoa all nutrient concentrations were increased by a factor of 4. The stand density was maintained at eight plants per m^2^ throughout. There were two CO_2_ treatments, 400 and 600 μmol·mol^−1^, each ±20 μmol·mol^−1^ maintained 24 h per day by the injection of pure CO_2_ or CO_2_-free air under the control of WMA-4 or WMA-5 infrared CO_2_ analyzers, which sampled chamber air continuously. When the first mainstem flowers had reached anthesis for a given cultivar, half of the plants of that cultivar were placed in other chambers with day/night temperatures of 35/29 °C, with a dew point temperature of 25 °C for 7 days. This high temperature treatment was chosen based on our observation of poor field yields of Cherry Vanilla in Beltsville, where those temperatures are typical of summer hot spells. The initial CO_2_ treatments were maintained during the high temperature treatments. After the high temperature treatments, the plants were returned to the original growth conditions, and all plants were grown to seed maturity. 

Leaf photosynthesis and stomatal conductance were measured before, during, and after the high temperature treatments, using a CIRAS-3 portable photosynthesis system with CO_2_, light, temperature, and humidity control (PP Systems, Amesbury, MA, USA). On each growth stage, for each replicate chamber run, three leaves from different plants per cultivar per treatment were measured. Measurements were made on upper canopy leaves which were fully expanded and fully exposed to light. Measurements were made near midday at the current daytime growth temperature (either 20 or 35 °C), at 400 or 600 μmol·mol^−1^ CO_2_ concentrations, at the daytime growth PPFD of 1000 μmol·m^−2^ s^−1^. For plants grown at the lower CO_2_, photosynthesis measurements were also made at the higher CO_2_, to assess the short-term response. The water vapor pressure inside the leaf cuvette was set to match that corresponding to the current growth dew point temperature (either 10 or 25 °C).

A total of eight different controlled environment chambers were utilized, with treatments randomly assigned to chambers over a span of 24 months. There were three replicate chambers per treatment per cultivar, with five or six plants per treatment replicate per cultivar. At seed maturity, the aboveground biomass of each plant was separately dried at 70 °C in a forced air oven, and seed and stem dry mass were separated and weighed. Any remaining leaf material was discarded. Harvest index was calculated as the seed dry mass divided by the total seed and stem dry mass. Analysis of variance was conducted using the three chamber replicates of each treatment and cultivar. Because ANOVA indicated a significant Cultivar × CO_2_ × Temperature interaction (*p* = 0.023) for seed mass, the CO_2_ and temperature treatments were analyzed separately for each cultivar.

## Figures and Tables

**Table 1:** Photosynthesis (in μmol CO_2_ m^−2^·s^−1^) of upper leaves of three cultivars of quinoa grown at ambient (400 μmol·mol^−1^) or elevated (600 μmol·mol^−1^) CO_2_ concentrations, with or without a high temperature stress treatment during anthesis. Photosynthesis was measured at the growth PPFD of 1000 μmol·m^−2^·s^−1^, and at the current daytime growth temperature (20 °C before and after the stress, and 35 °C during the stress). Photosynthesis was measured at the growth CO_2_, ambient or elevated, and ambient plants were also measured at elevated CO_2_ (ambient at elevated). Numbers followed by the same letters within a growth stage were not significantly different at *p* = 0.05.

Growth Stage	Cultivar	Ambient	Elevated	Ambient at Elevated
Before stress	Cherry Vanilla	38.8a	42.6b	44.2b
	Red Head	39.5a	42.5b	45.0b
	Salcedo	37.8a	43.6b	42.0b
During stress	Cherry Vanilla	32.7a	39.8b	40.8b
Stressed plants	Red Head	34.1a	41.6b	41.7b
	Salcedo	32.2a	37.8b	38.9b
After stress	Cherry Vanilla	35.2a	34.2a	40.3b
Stressed plants	Red Head	36.3a	37.1a	41.1b
	Salcedo	34.1a	35.0a	39.6b
After stress	Cherry Vanilla	34.7a	35.4a	40.8b
Control plants	Red Head	35.8a	36.2a	41.0b
	Salcedo	35.1a	35.8a	39.3b
